# Efficient extraction of small and large RNAs in bacteria for excellent total RNA sequencing and comprehensive transcriptome analysis

**DOI:** 10.1186/s13104-015-1726-3

**Published:** 2015-12-08

**Authors:** Rajandas Heera, Parimannan Sivachandran, Suresh V. Chinni, Joanne Mason, Larry Croft, Manickam Ravichandran, Lee Su Yin

**Affiliations:** Department of Biotechnology, Faculty of Applied Sciences, AIMST University, Semeling, 08100 Bedong, Kedah Malaysia; Unit of Biochemistry, Faculty of Medicine, AIMST University, Semeling, 08100 Bedong, Kedah Malaysia; Malaysian Genomics Resource Centre, 27-9, Level 9 Boulevard Signature Offices, 59200 Mid Valley City, Malaysia; Oxford Biomedical Research Centre, Old Road Headington Oxford, Oxfordshire, OX3 7LE UK

**Keywords:** Bacterial RNA extraction, DNase I treatment, RIN, RNA-Seq, Small and large RNAs, Transcriptome coverage, Reproducible

## Abstract

**Background:**

Next-generation transcriptome sequencing (RNA-Seq) has become the standard practice for studying gene splicing, mutations and changes in gene expression to obtain valuable, accurate biological conclusions. However, obtaining good sequencing coverage and depth to study these is impeded by the difficulties of obtaining high quality total RNA with minimal genomic DNA contamination. With this in mind, we evaluated the performance of Phenol-free total RNA purification kit (Amresco) in comparison with TRI Reagent (MRC) and RNeasy Mini (Qiagen) for the extraction of total RNA of *Pseudomonas aeruginosa* which was grown in glucose-supplemented (control) and polyethylene-supplemented (growth-limiting condition) minimal medium. All three extraction methods were coupled with an in-house DNase I treatment before the yield, integrity and size distribution of the purified RNA were assessed. RNA samples extracted with the best extraction kit were then sequenced using the Illumina HiSeq 2000 platform.

**Results:**

TRI Reagent gave the lowest yield enriched with small RNAs (sRNAs), while RNeasy gave moderate yield of good quality RNA with trace amounts of sRNAs. The Phenol-free kit, on the other hand, gave the highest yield and the best quality RNA (RIN value of 9.85 ± 0.3) with good amounts of sRNAs. Subsequent bioinformatic analysis of the sequencing data revealed that 5435 coding genes, 452 sRNAs and 7 potential novel intergenic sRNAs were detected, indicating excellent sequencing coverage across RNA size ranges. In addition, detection of low abundance transcripts and consistency of their expression profiles across replicates from the same conditions demonstrated the reproducibility of the RNA extraction technique.

**Conclusions:**

Amresco’s Phenol-free Total RNA purification kit coupled with DNase I treatment yielded the highest quality RNAs containing good ratios of high and low molecular weight transcripts with minimal genomic DNA. These RNA extracts gave excellent non-biased sequencing coverage useful for comprehensive total transcriptome sequencing and analysis. Furthermore, our findings would be useful for those interested in studying both coding and non-coding RNAs from precious bacterial samples cultivated in growth-limiting condition, in a single sequencing run.

**Electronic supplementary material:**

The online version of this article (doi:10.1186/s13104-015-1726-3) contains supplementary material, which is available to authorized users.

## Background

Bacteria play major roles in our everyday lives in more ways than one can imagine. Depending on the environment, bacteria can be detrimental by being causative agents for various diseases [1, 2] or beneficial by synthesizing value-added products [3] and performing bioremediation of contaminated sites [4]. However, to identify what confers the pathogenicity and/or ability to synthesize value-added products, one must know the changes at the metabolic level and understand the underlying molecular mechanisms in the bacterial cells.

Obtaining these valuable biological and molecular insights into the transcriptome of bacterial cells is now possible with the introduction of next-generation sequencing (NGS) platforms that perform massive parallel sequencing. NGS for transcriptomics often referred to as RNA-Seq has tremendously increased transcriptome coverage, which in turn has enabled the discovery of novel non-coding RNAs (ncRNA), untranslated regions (UTRs) and rare transcript variants [5–7]. In fact, RNA-Seq has now become the “gold standard” for annotation of transcripts and differential gene expression analysis as the output can be efficiently mapped to the genome and the expression of each transcript can be quantified by digitally recording how frequently they are represented in a sequenced sample [8–10].

However, obtaining good sequencing coverage for an accurate representation of the transcriptome is impeded by the difficulties of isolating total RNA with good yield, high integrity and minimal genomic DNA contamination. Owing to the short half lives and sensitive nature of bacterial RNAs, much care has to be taken to extract high quality RNA with minimal degradation that are suitable for sequencing [11–13]. In recent years, many commercial total RNA extraction kits employ spin-column technology and organic solvents to overcome those challenges [14–19]. Although these kits promise pure total RNA extracts, co-extraction of genomic DNA seems unavoidable [20]. Researchers working on high-throughput sequencing methods such as RNA-Seq therefore have to perform additional DNase I treatment to remove contaminating DNA prior to cDNA conversion. In addition, most literature seems to undermine the importance of the DNase I treatment by providing limited information on the treatment and the method for removing the contaminating DNA [17, 18, 21, 22].

Aside from these, till date, no study has identified the best kit to obtain total bacterial RNA comprising high and low molecular weight transcripts with minimal genomic DNA contamination that is compatible with RNA-Seq. While Fromm et al. [23] had shown that the Phenol-free total RNA purification kit (Amresco, USA) is capable of yielding low and high molecular weight transcripts from an ectoparasite, the suitability and performance of these RNA extracts were not evaluated using RNA-Seq. Determining the performance of Phenol-free RNA extracts in terms of transcriptome coverage and depth would especially benefit researchers who are interested in studying the expression of high and low abundance functional (mostly large mRNAs) and regulatory RNAs (often small RNAs) in a single sequencing run.

Furthermore, we had previously shown using Fourier Transform Infrared coupled Attenuated Total Reflectance (FTIR-ATR) spectroscopy that a strain of *Pseudomonas aeruginosa* (AIMST H2) was able to degrade polyethylene (PE) under laboratory conditions [24]. We were therefore interested in studying both the coding genes (mRNAs) and non-coding genes (ncRNAs) that are expressed during the PE biodegradation process using RNA-Seq. With these in mind, we were keen to evaluate the performance of Phenol-free total RNA purification kit (Amresco, USA) for RNA extraction from *P. aeruginosa*, the model organism used in this study. In addition, the performance of Phenol-free kit was evaluated in comparison with other total RNA extraction kits/reagents widely used in previous studies for RNA-Seq, TRI Reagent (Molecular Research Centre, MRC, USA) and RNeasy Mini kit (Qiagen, USA) [15, 18, 19, 25]. All three methods were evaluated in terms of the RNA quality, yield, DNA contamination and ability to consistently isolate both high and low molecular weight transcripts. Subsequently, the RNA samples obtained using the best extraction method were sequenced using the Illumina HiSeq 2000 system and the quality of the sequencing data in terms of the coverage, depth and reproducibility obtained were assessed using bioinformatic analyses.

## Methods

### Growth and preparation of *P. aeruginosa* AIMST H2 culture

Ten ml of Luria–Bertani (LB) culture of *P. aeruginosa* AIMST H2 was inoculated into a Erlenmeyer flask containing 100 ml minimal medium supplemented with 0.2 % (w/v) glucose (control) or 0.25 g PE powder as the sole carbon source. Once inoculated, the flasks were placed in a 37 °C shaking incubator and left to agitate at 180 rpm until the growth reached mid-logarithmic phase. The bacteria titer in each flask was adjusted to 1 × 10^9^ cells to standardize the number of cells subjected to RNA extraction. The cells were harvested by centrifugation at 10,000 rpm for 10 min and the supernatant was discarded. The resulting cell pellet was maintained on ice and processed immediately to minimize RNA degradation.

### RNA extraction

RNA extraction was done with cell pellets containing 1 × 10^9^ cells from three independent biological replicates of each condition using three different kits/reagent: TRI Reagent, RNeasy Mini kit and Phenol-free total RNA purification kit.

### TRI reagent

Total RNA extraction was performed according to the manufacturer’s protocol with slight modification. One tenth volume of 3 M sodium acetate (pH 5.2) and three volume of absolute ethanol were used instead of isopropanol for precipitating RNA. This reaction mixture was incubated at −80 °C for 2 h to allow efficient precipitation of RNA. After washing the RNA pellet with 75 % ice cold ethanol, it was reconstituted with 50 µl of nuclease-free water. The RNA elutes were stored at −80 °C till further use.

### RNeasy mini kit and phenol-free total RNA purification kit

The cell pellet was reconstituted with 400 µl of sterile minimal media. Then, 800 µl RNAprotect Bacteria Reagent (Qiagen, USA) was added and the contents in the tube were vortexed prior to a 5 min incubation at room temperature (RT). The tubes were centrifuged at 5000×*g* for 10 min. The supernatant was discarded and the cell pellet was subjected to total RNA extraction and on-column DNase I treatment according to the manufacturers’ protocol.

### Removal of contaminating genomic DNA by DNase I digestion

Total RNA obtained from replicates of each extraction method was subjected to PCR using bacterial 16S rDNA primers, Bak11-W (5′-AGTTTGATCMTGGCTCAG-3′) and Bak-R (5′-GGACTACHAGGGTATCTAAT-3′), to determine the presence of contaminating genomic DNA in the extracts [26]. PCR reactions were performed in 20 μl volumes containing 100 ng of RNA, 1× PCR buffer containing 750 mM Tris–HCl (pH 8.8 at 25 °C), 200 mM (NH_4_)_2_ SO_4_, 0.1 % Tween 20; 2.5 mM MgCl_2_; 0.16 mM dNTP mix; 20 pmol of Bak11-W and Bak-R primers and 0.75 U Taq DNA polymerase (Fermentas, Lithuania). Amplification was performed with an initial denaturation at 95 °C for 3 min, followed by 30 cycles of denaturation at 95 °C for 30 s, annealing at 52 °C for 30 s and an extension at 72 °C for 30 s. A final extension step was also included at 72 °C for 5 min. Based on the PCR analysis, additional DNase I treatment was performed on the RNA extracts with contaminating genomic DNA to remove the residual DNA. One hundred µl reaction consisting of 50 µl RNA elutes, 10 U DNase I (Fermentas, USA), 1× DNase I buffer (Fermentas, USA), 80 U recombinant RNasin ribonuclease inhibitor (Promega, USA) and RNase-free water was incubated at 37 °C for an hour. After digestion, the RNA was recovered from the DNase I reaction mixture using phenol:chloroform, low pH (pH 4.0, Amresco, USA) extraction followed by ethanol precipitation. The RNA pellet was reconstituted with 50 µl of nuclease-free water before 100 ng of the RNA wassubjected to PCR analysis again to determine the presence of genomic DNA. The additional DNase I treatment was repeated until no residual DNA was detected via PCR in the RNA samples. Purity of the RNA extracts was also determined before and after the DNase I treatment using spectrophotometric analysis.

### Determination of RNA yield and integrity

The RNA concentration and quality was determined using the RNA Nano 6000 LabChip kit (Agilent Technologies, USA). The LabChips were run in an Agilent 2100 Bioanalyzer following the manufacturer’s instructions. Aside from quantifying, this method determines the RNA Integrity Number (RIN) of an RNA sample, which indicates the overall integrity with a score of 1 indicating degradation of RNA and a score of 10 indicating intact RNA [27].

### Next-generation sequencing (RNA-Seq)

Four purified RNA extracts (2 from cells grown in glucose supplemented medium and another 2 from cells grown in PE supplemented medium) with the best RIN values were selected for sequencing. First, ribosomal depletion was performed using Ribo-Zero Magnetic Kit (Epicentre, USA). Then cDNAs were synthesized using TruSeq RNA Sample Preparation Kit (Illumina, USA) and SuperScript II Reverse Transcriptase (Invitrogen, USA). A minimum of 20 ng cDNA was fragmented using Covaris S220 (Covaris Inc., USA) to a targeted size of <500 bp. The fragmented cDNA were then end-repaired, ligated to Illumina TruSeq Adapters and PCR-enriched using TruSeq RNA Sample Preparation Kit (Illumina, USA). The final sequencing libraries were quantified using KAPA kit (KAPA Biosystem, USA) on a Stratagene Mx-3005P qPCR system (Agilent Technologies, USA). Library sizes were confirmed using Agilent Bioanalyzer High Sensitivity DNA Chip (Agilent Technologies, USA). The resulting libraries were subjected to cluster generation and sequenced using an Illumina flow cell, 202 cycles (101 bp paired-end reads) on the Illumina HiSeq 2000 system (Illumina, USA). All steps were performed according to the manufacturers’ protocol, unless otherwise stated.

### Bioinformatic analysis

The sequences that correspond to the Illumina sequencing adapters and low-quality fastq reads were trimmed and filtered using Trimmomatic v0.32 [28]. The processed output files for all 4 samples were then analyzed using FastQC v0.10.1 to validate the quality of the fastq reads. Reference-based alignment of the processed reads was performed individually for all 4 samples with Bowtie 2 v2.1.0. *P. aeruginosa* PAO1 strain (GenBank ID: AE004091) was used as the reference genome for the alignment. HTSeq-count was performed on each alignment file to obtain the count table for each sample. The count table features the number of paired reads that map to each gene as specified in the generic feature format (gff) file of the reference genome. Finally, differential gene expression analysis was performed using DESeq with the count table generated from all four samples.

## Results and discussion

### Removal of contaminating genomic DNA by DNase I digestion

Genomic DNA is a concern especially for bacterial RNA sequencing as the cDNAs corresponding to the contaminating genomic DNA will result in inaccurate representation of the expressed transcripts [17]. Since DNA is usually co-extracted during the RNA isolation procedures, evaluating the presence of contaminating genomic DNA and removal of these molecules using DNase I treatment is a must for RNA samples prior to sequencing. In this study, we performed DNase I treatment followed by low pH phenol:choloroform extraction for removing the degraded contaminating genomic DNA. Low pH phenol:choloroform RNA extraction was performed instead of heat inactivation of DNase I to avoid subjecting the RNA extracts to heat which can cause RNA degradation [29]. PCR amplifications were performed on the RNA extracts before and after the in-house DNase I treatment to detect genomic DNA contamination. The PCR analysis prior to the in-house DNase I treatment revealed that 100 % of the reactions had strong amplification of the 16S rDNA gene, indicating the presence of genomic DNA contamination in RNA extracts of all three methods. Following the in-house DNase I treatment, PCR analysis revealed that there was no detectable genomic DNA contamination in the RNA extracts obtained using RNeasy and Phenol-free kits. However, 50 % of TRI Reagent extracts still expressed faint bands that imply the presence of low amounts of genomic DNA, requiring additional DNase I treatment. The residual genomic DNA in these extracts were then removed by performing another additional DNase I treatment, which was sufficient to ensure that there were no detectable DNA during PCR verification. Assessment of RNA extracts yielded from all three methods using UV spectrophotometry, on the other hand, indicated improvement in purity (A260/A280) after the in-house DNase I treatment, from the initial purity of 1.6–1.9 to 1.9–2.0 (Table 1).

**Table 1:** Summary of results from RNA extraction performed with the indicated methods

Method/kit	Total RNA yield (µg)			RIN value	Purity (A260/A280)
	Glucose	PE	Average		
TRI reagent (MRC, USA)	2.83 ± 0.95	2.63 ± 0.25	2.72 ± 0.58	NA	1.94 ± 0.01
RNeasy Mini kit (Qiagen, USA)	6.80 ± 0.20	2.15 ± 0.10	4.48 ± 2.69	9.65 ± 0.24	1.98 ± 0.05
Phenol-free total RNA purification kit (Amresco, USA)	26.55 ± 2.22	14.72 ± 2.23	20.63 ± 7.07	9.85 ± 0.20	2.01 ± 0.03

Total yield indicated from starting cell density of 10^9^ bacterial cells. NA-not available

The aforementioned observations also indicated that the protocols recommended by the manufacturers including the on-column DNase I treatments applied for the RNeasy and Phenol-free kit extracts did not yield DNA-free RNA and the in-house DNase I treatment was necessary to remove the contaminating genomic DNA. Our findings coincided with that of Schwochow et al. [20], who also found that the DNase I treatment suggested by the manufacturers were insufficient and the RNA extracts required additional enzymatic treatment to remove the remaining genomic DNA. The in-house DNase I treatment is therefore necessary to improve the RNA purity by eliminating contaminating genomic DNA, which is an essential requirement for RNA-Seq.

### RNA yield and integrity

**Fig. 1:**
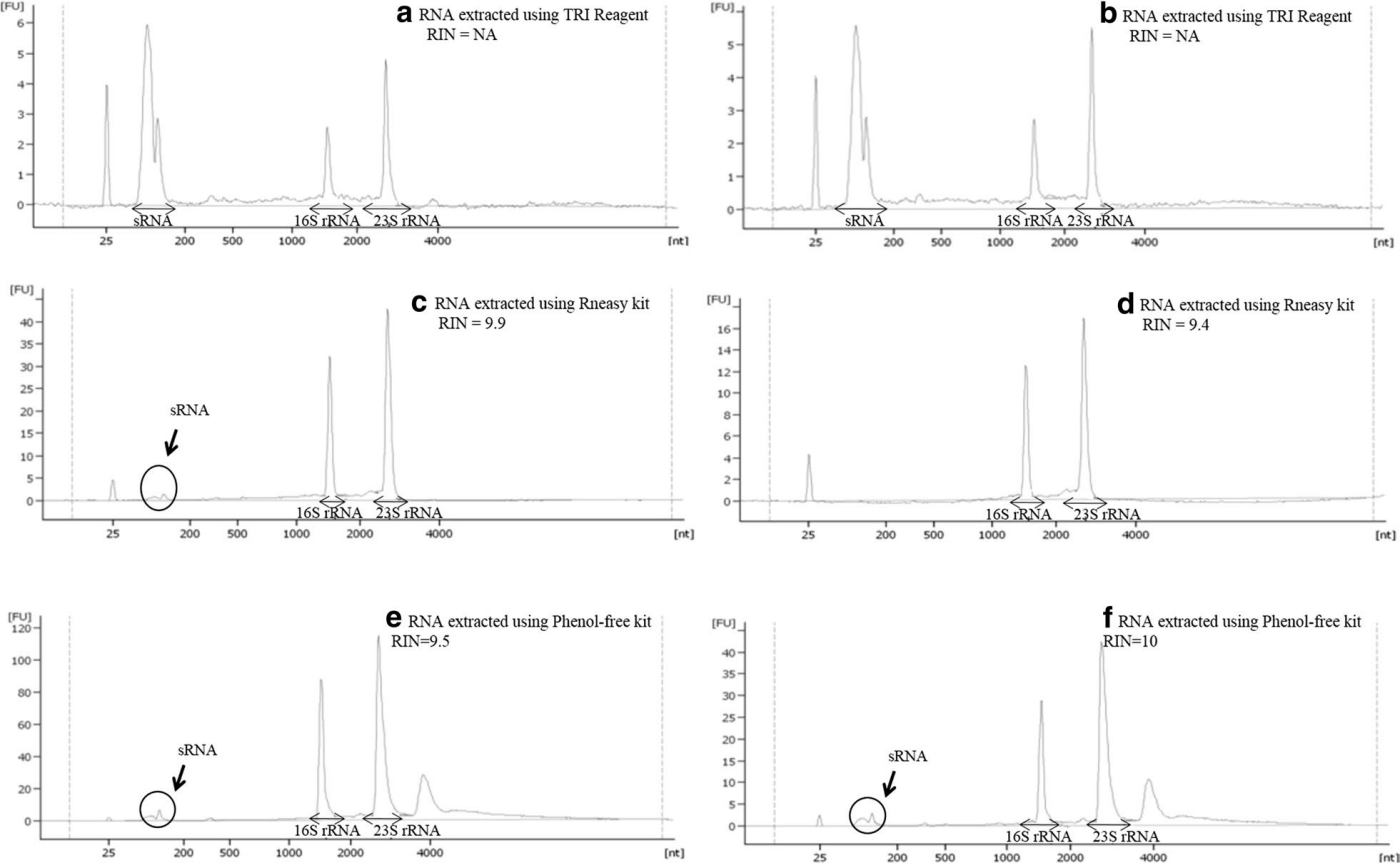
Electropherogram of RNA elutes obtained using three different RNA extraction methods. **a** RNA extracted from glucose-supplemented cells using TRI Reagent; **b** RNA extracted from PE-supplemented cells using TRI Reagent; **c** RNA extracted from glucose-supplemented cells using RNeasy kit; **d** RNA extracted from PE-supplemented cells using RNeasy kit; **e** RNA extracted from glucose-supplemented cells using Phenol-free kit; **f** RNA extracted from glucose-supplemented cells using Phenol-free kit. *NA* not available

Following the in-house DNase I treatment, total RNA concentrations and integrity of all RNA extracts were determined using RNA Nano 6000 LabChip kit. The yield and RIN values of RNA extracts from the respective extraction method are shown in Table 1, while the representative electropherograms are shown in Fig. 1. The Phenol-free kit extracts had the highest RNA concentration (20.63 ± 7.07 µg) when compared to RNeasy kit (4.48 ± 2.69 µg) and TRI Reagent (2.72 ± 0.58 µg), which recorded almost ten times lower yield. Further analysis using one-way ANOVA test also revealed that the Phenol-free kit had significantly higher yield (*p* < 0.05) as compared to the other two kits. Apart from that, it was observed that the average RNA yield was consistently lower in extracts obtained from PE-supplemented cells compared to those from glucose-supplemented cells. This could have been due to the lower number of genes being transcribed in cells grown under stressful/growth-limiting condition as a result of 6S RNA gene up-regulation (observed in the output of the differential gene expression analysis). Bacterial 6S RNA inhibits transcription of genes when the cell is undergoing stress by specifically binding to the RNA polymerase holoenzyme containing sigma70 that controls the expression of most genes under the normal growth conditions. This form of regulation helps redirect the bacterial cells to an alternative survival strategy when grown under growth-limiting/stressful conditions [30, 31].

It is also important to note that the overall yields obtained for the RNeasy and Phenol-free kits were lower than that expected (according to manufacturer). The reduced yield could be accounted for by the competitive binding of the contaminating genomic DNA that may have reduced the amount of RNA capable of binding to the silica membrane. This was evident as the initial RNA extracts prior to the in-house DNase I treatment had detectable genomic DNA contamination. The reduced yield in TRI Reagent extracts, on the other hand, could have been due to inefficient bacterial cell lysis as the extraction method completely relied on guanidium thiocyanate, unlike both the kits which utilized lysozyme aside from the cell lysis buffer provided. However, the reduced yield is not a major issue as far as RNA-Seq is concerned as the sample preparation procedure only requires 0.1–4 µg of RNA as starting material.

Aside from this, the Phenol-free kit RNA extracts also had the highest RNA integrity with almost perfect RIN value of 9.85 ± 0.20. This was followed by the RNeasy kit extracts that recorded RIN value of 9.65 ± 0.20. Our finding coincided with a study by Rump et al. [32] which yielded almost similar RIN values (9.57 ± 0.59) using RNeasy kit in extracting DNA-free RNA from *Salmonella* cells. On the contrary, RNA samples extracted using TRI Reagent failed to generate RIN values due to the overwhelming amount of small RNAs (sRNAs) as shown by the sharp peak between 25 and 200 bases (Fig. 1a, b).

### Size distribution of the RNA species

Apart from these, we observed some variation in the yield of low molecular weight RNA among the three RNA extraction methods. TRI Reagent seemed very efficient in extracting sRNAs, as shown by the sharp peak between 25 and 200 bases in the electropherogram (Fig. 1a, b), with relatively low amounts of high molecular weight RNAs. In contrary, RNeasy kit proved to be excellent in isolating high molecular weight RNAs with only one of its extracts containing traces of sRNAs (Fig. 1d). The Phenol-free kit which gave the highest total RNA yield and RIN value, also consistently yielded total RNA with good ratio of low and high molecular weight RNA species. This RNA profile was similar to that observed by Fromm et al. [23], in which they compared the efficiency of Phenol-free kit with 5 other kits for RNA extraction of an ectoparasite, *Gyrodactylus salaris.* They found that the RNA extracts isolated using Phenol-free kit yielded the highest total RNA yield with good amounts of microRNAs.

However, when the amounts of small RNA was quantified with reference to fluorescence intensity, minimal difference was observed between the sRNA peaks of TRI Reagent extracts and Phenol-free extracts. Since TRI Reagent extracts were enriched in low molecular weight RNAs, it can be used for studies which exclusively focus on sRNA (which includes ncRNA). In addition, the low RNA yield obtained using TRI Reagent can be improved by up-scaling the culture volume as this technique has no limitations in terms of nucleic acid binding capacity posed by spin-column techniques. RNeasy kit, on the other hand, proved to be suitable for researchers interested in studying mRNAs as the extracts contained good quality high molecular weight transcripts. Since our group’s interest was to study both mRNAs and ncRNAs expressed in the bacteria in a single sequencing run, the RNA extracts obtained using Phenol-free kit were the most suitable as they had good ratio of low and high molecular weight transcripts, with high yield and integrity.

### Transcriptome sequencing coverage and depth

Sequencing was performed on four samples (duplicates for each condition) extracted using Phenol-Free kit coupled with in-house DNase I treatment. A summary of RNA yield, purity and RIN values of the samples is shown in Table 2. All 4 samples had sufficiently high concentrations of RNA (>8 µg), high purity (A260/A280 ~2.0) and high RIN values of above 9 (>8 is required for sequencing), which indicated good quality RNA [33].

**Table 2:**
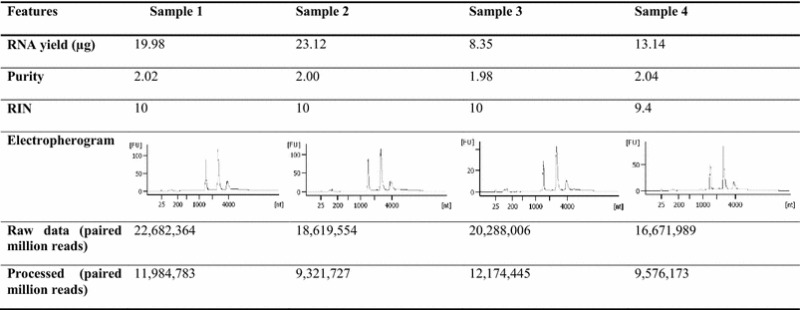
Summary of sequenced transcriptome samples

RNA yield was quantified using Qubit RNA. The processed data for all four samples had PHRED quality scores of 25 and above

Sample 1, glucose 1; Sample 2, glucose 2; Sample 3, PE 1; Sample 4, PE 2

Each RNA sample which were converted to cDNAs and sequenced yielded approximately 16–22 million paired end reads of raw data. Upon pre-processing the data to remove low quality bases using Trimmomatic, approximately 9–12 million paired end reads were retained. The average PHRED quality scores for all four samples increased from 15 to 25, which made them suitable for analysis as typically, PHRED quality scores of 20 and above are preferred [34].

Reference-based alignment and differential gene expression analysis were then performed using Bowtie 2 and DESeq, respectively. Based on the output of reference-based alignment, the overall sequencing coverage of the samples were excellent. Using the *P. aeruginosa* PAO1 genome as Ref. [35], we were able to detect the expression of 5435 out of 5574 (97.5 %) coding genes in the *P. aeruginosa* AIMST H2 transcriptome data. The expression of 28 out of 30 sRNAs previously documented in the *P. aeruginosa* PAO1 genome were also detected.

**Fig. 2:**
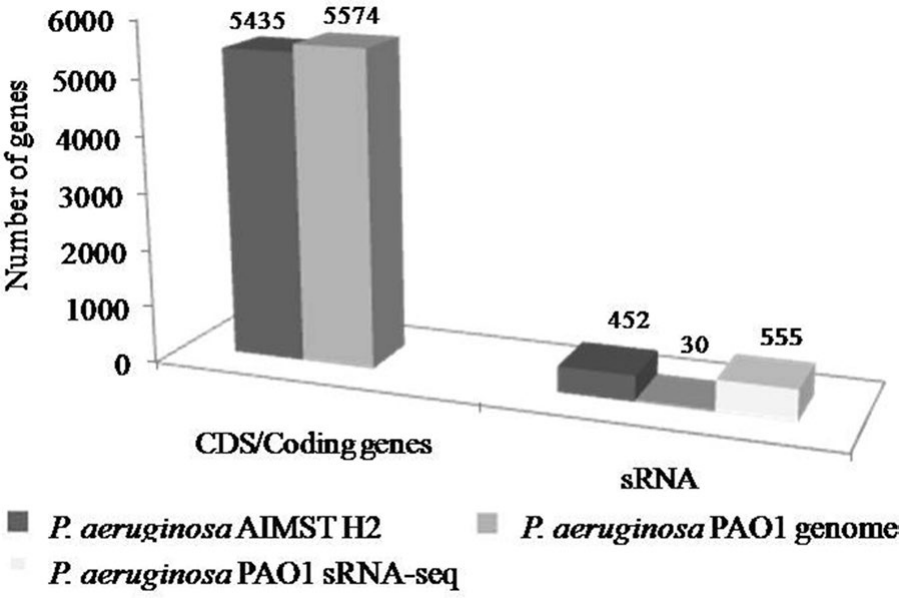
Overall sequencing coverage of *P. aeruginosa* AIMST H2 transcriptome with reference to *P. aeruginosa* PAO1 genome and *P. aeruginosa* PAO1 sRNA-seq

In addition, visual screening of the reference-based alignment output via Artemis Genome browser revealed expression ‘peaks’ in many intergenic regions which lack annotation in the genome. Upon extended analysis, we found that 452 of these peaks in the intergenic regions coincided with the sRNAs discovered by Gómez-Lozano et al. [36] who performed an elaborate sRNA sequencing (sRNA-Seq) study of *P. aeruginosa* PAO1 grown in LB broth [36]. These 452 intergenic sRNAs alone contributed to 81.4 % of 555 sRNAs identified by Gómez-Lozano et al. [36]. The remaining 139 coding genes and 103 sRNAs that were not detected were either not present in *P. aeruginosa* AIMST H2 genome or were present, but not transcriptionally active under the experimental growth conditions. Furthermore, a small number of these sRNAs which were not detected were antisense RNAs which overlapped with coding genes. Unfortunately, the expression of these antisense sRNAs could not be confirmed as we were not able to distinguish the expression of the coding genes from that of the antisense sRNAs due to the nature of the non-strand-specific sequencing performed. Therefore, repeating transcriptome sequencing with a directional library prepared using the same RNA extracts would solve this problem and enhance the discovery of overlapping anti-sense transcripts. An illustration of the overall transcriptome sequencing coverage with reference to *P. aeruginosa* PAO1 genome [35] and *P. aeruginosa* PAO1 sRNA-Seq [36] is shown in Fig. 2.

**Fig. 3:**
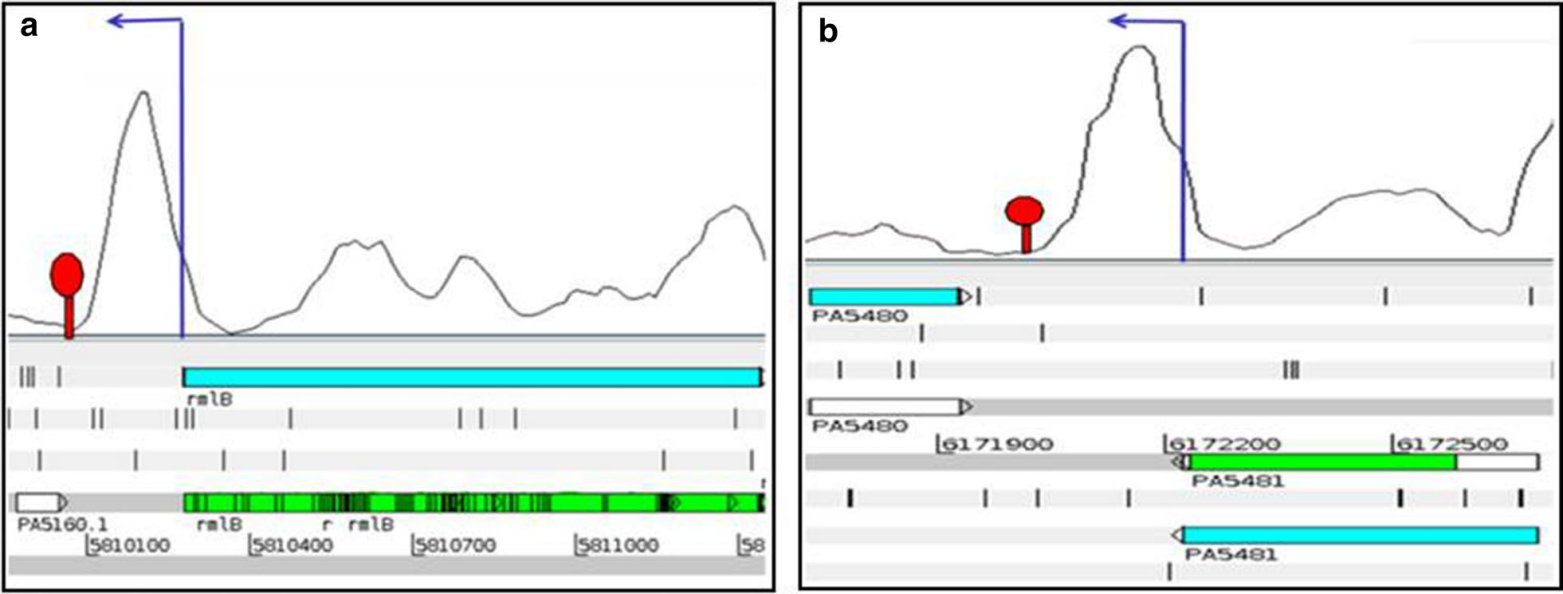
Potential novel sRNAs in *P. aeruginosa*. **a**, **b** Refer to the potential novel sRNAs observed and their coordinates according to *P. aeruginosa* PAO1 genome. The *dark blue arrows* indicate transcription start sites (TSS) and the red stem-loop structures indicate terminator

Interestingly, we also observed expression “peaks” at seven other intergenic regions in the *P. aeruginosa* transcriptome which have not been categorized as sRNAs in previous studies. All 7 regions have transcription start sites (TSS) and rho-independent terminators which were determined using Dötsch et al. [19] and TranstermHP. RFAM, BLASTn and BLASTx analysis of these intergenic sequences revealed that they do not code for any known RNAs or proteins, suggesting discovery of potential novel intergenic sRNAs. Representative images of these potential novel intergenic sRNA transcripts are illustrated in Fig. 3.

**Fig. 4:**
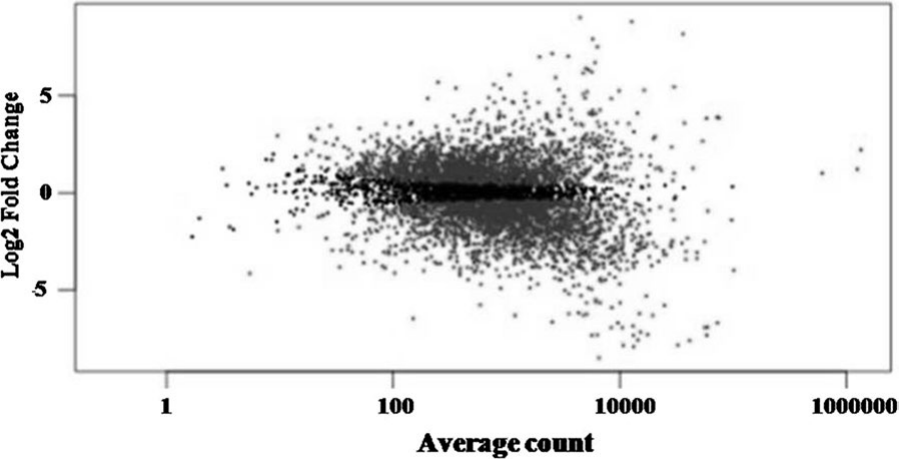
MA plot. *Grey dots* denote significantly differentially expressed genes, while *black dots* denote non-differentially expressed genes. *Dots* with a positive log2 fold change indicate genes which were up-regulated in the PE supplemented bacterial cells and dots with a negative log2 fold change indicate genes which were down-regulated in the PE supplemented bacterial cells

Apart from these observations that demonstrated excellent transcriptome coverage, the output of the differential gene expression analysis was analyzed to examine the dynamic range of transcription in *P. aeruginosa*. The MA plot obtained from the preliminary analysis (*p* < 0.05) showed that a majority of the expressed genes had a mean read count between 100 and 10,000 with log2 fold change between −2.5 and 2.5 (Fig. 4). Fewer genes were expressed at log2 fold change above 3 and below −3. Intriguingly, approximately 507 genes with counts below 100 were observed. These low abundance transcripts are not present by chance as their counts are consistent across replicates from the same conditions. Consistent counts were also observed in genes with higher counts as shown in the list provided in the Additional file 1: Table S1. It was also noted that these counts are consistent across the biological replicates that were sequenced. These observations clearly demonstrate the reproducibility and consistency of the RNA extraction technique.

**Fig. 5:**
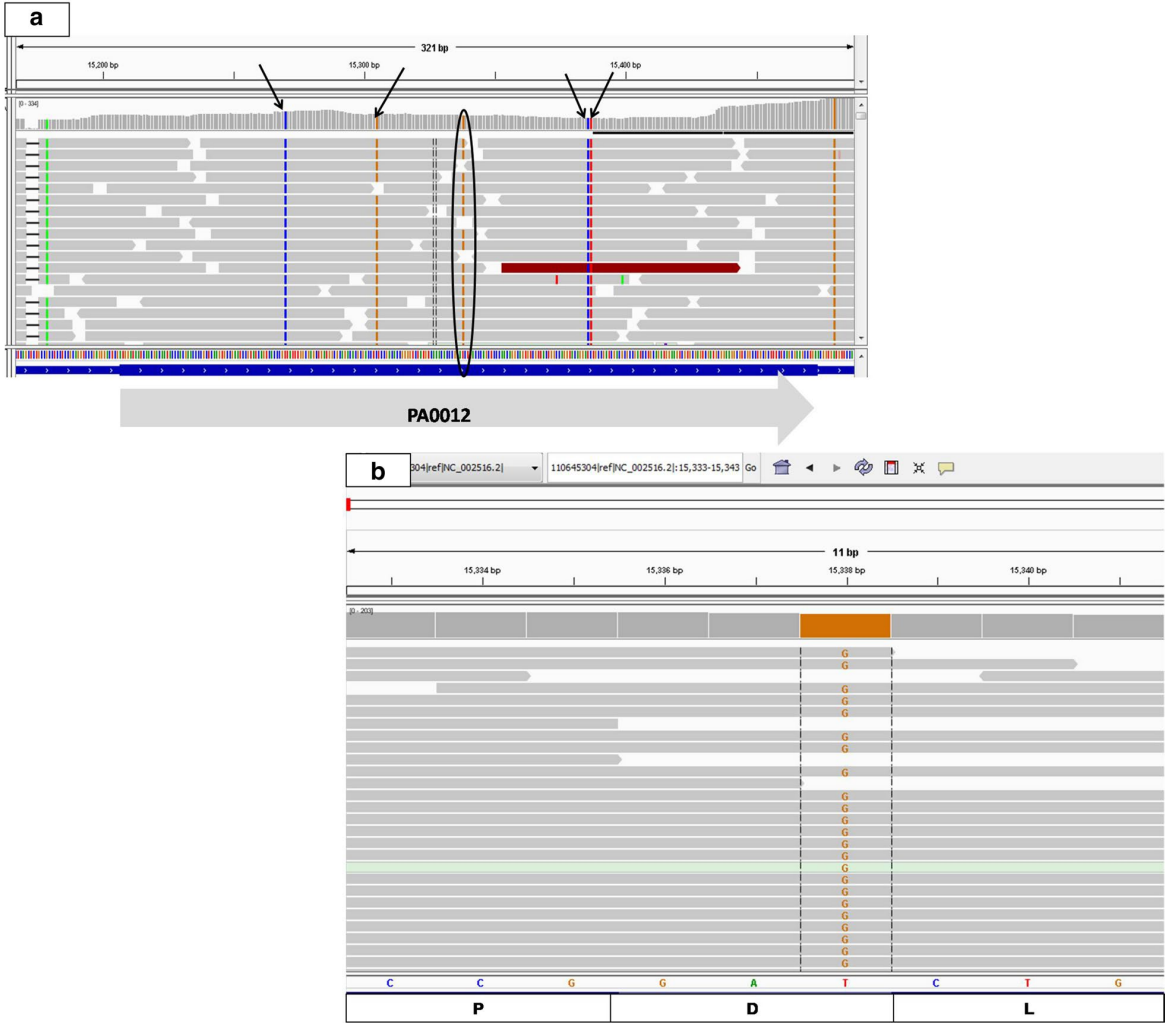
SNPs observed in gene PA0012. **a** All *four black arrows* point to synonymous base substitutions, while the *circle* points to an observed non-synonymous substitution; **b** zoomed in view of the non-synonymous substitution in the last base of the codon

Further analysis of the *P. aeruginosa* AIMST H2 transcriptome also revealed the presence of genome-wide single nucleotide polymorphisms (SNPs) when aligned against *P. aeruginosa* PAO1 strain as the reference genome. An example of the observed SNPs is illustrated in Fig. 5. Five SNPs were observed in gene PA0012 which codes for a protein with a pilus assembly protein (PilZ) domain. The SNP/mutation (base substitution) in all five codons were consistent across all the reads that aligned to the gene. Four of them (shown using black arrows in Fig. 5a) resulted in synonymous codon substitution; i.e. the base substitution/mutation did not change the amino acid sequence of the protein, while 1 of them (circled in Fig. 5a and a zoomed in view is shown in Fig. 5b) resulted in a non-synonymous codon substitution. The mutation observed in Fig. 5b) involved substitution of the last base of the codon that resulted in change in amino acid, from aspartic acid, D to glutamic acid, E. Observation of SNPs is evidence of the sensitivity of the sequencing data obtained to detect mutation or change at a single base. This would be useful in identifying gene variants, especially when the alignment of transcripts are performed using closely related genomes or genomes of similar species.

The excellent transcriptome coverage and depth as well as observation of genome-wide SNPs are evidence that high quality RNA with minimal DNA contamination results in good quality sequencing output, providing valuable insights into bacterial transcriptomes. One can expect equally good quality RNA extracts and transcriptome coverage with other Gram-negative as well as Gram-positive bacteria provided the cells are treated with the RNAprotect Bacteria Reagent and the cell lysis step is efficient. Incorporation of the RNAprotect Bacteria Reagent treatment is crucial to stabilize the RNA and prevent degradation as bacterial transcripts have short half lives. Once stabilized with the Reagent, bacterial gene expression will not undergo any changes and the downstream analysis will reflect the true gene expression in the cells. Cell lysis, on the other hand, can be enhanced by increasing the lysozyme treatment time as recommended by the manufacturer or by increasing the concentration of lysozyme.

### Relevance of the study

The primary findings of this study (i.e. the determination of the best extraction method) is useful not only to facilitate high-throughput total bacterial transcriptome sequencing, but also to perform other RNomics based experiments like RT-qPCR and microarray analysis. Since the total RNA extraction method demonstrated was also tested on *P. aeruginosa* grown in minimal media supplemented with PE as the sole carbon source, our findings will also be useful for RNA extraction from bacteria grown in growth-limiting or stressful environmental conditions which limits cell growth.

## Conclusions

**Fig. 6:**
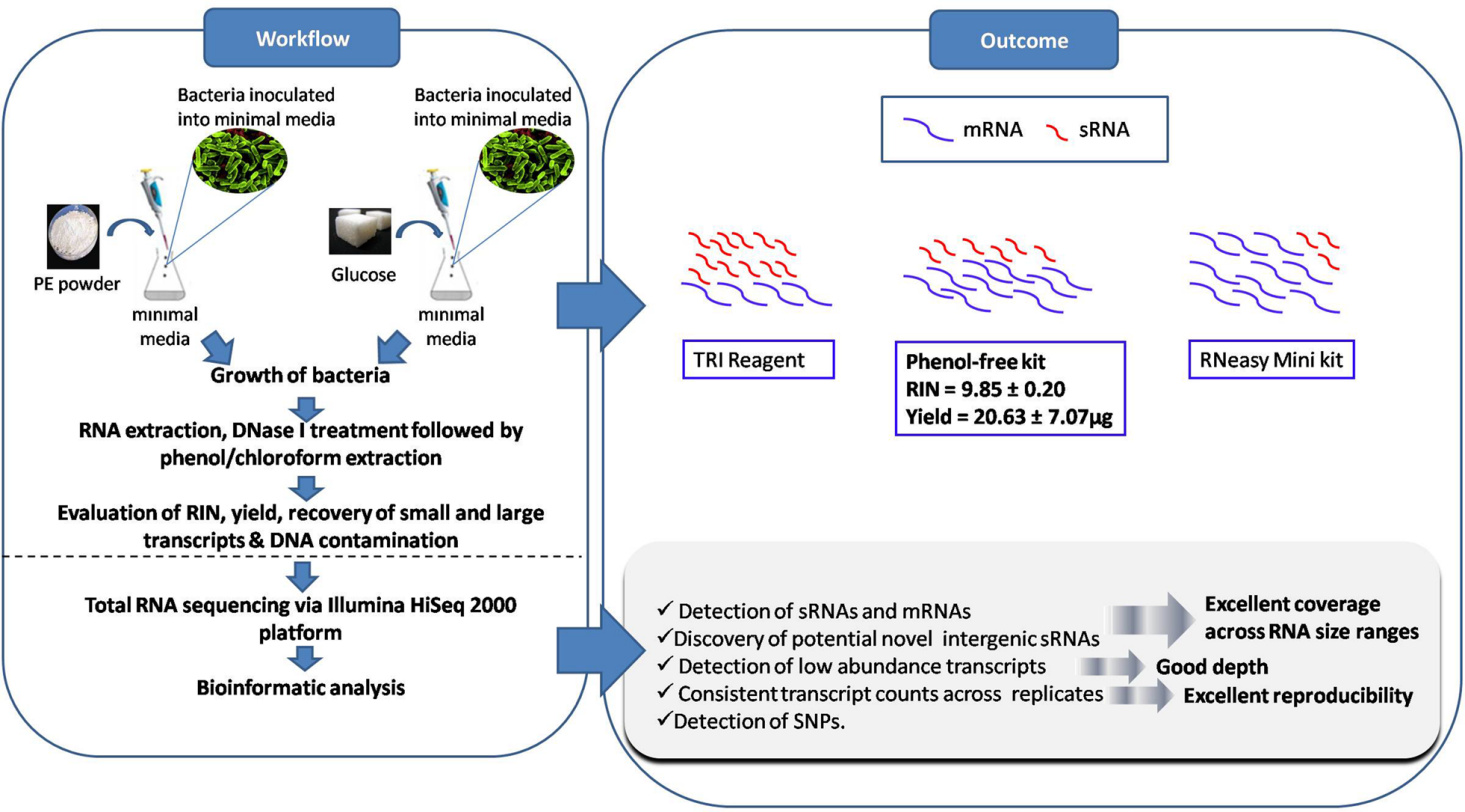
Schematic diagram illustrating an overview of the study

Based on the parameters assessed in this study, Amresco’s Phenol-free total RNA purification kit coupled with an in-house DNase I treatment yielded the best quality RNA extracts comprising low and high molecular weight transcripts with minimal DNA contamination from *P. aeruginosa*. Bioinformatic analysis of the transcriptome data obtained via sequencing of the Phenol-free RNA extracts revealed that the coverage and depth was good as a high percentage of the coding genes (97.5 %), sRNAs (81.4 %), seven potential novel intergenic sRNAs and more than 500 low abundance transcripts were successfully detected. Genome-wide SNPs were also observed in the *P. aeruginosa* AIMST H2 strain using the PAO1 strain as a reference. The sequencing coverage and depth demonstrated via the bioinformatic analysis clearly proved that the extraction method is suitable for researchers interested in studying both bacterial mRNAs and sRNAs in a single sequencing run. This method will also benefit those dealing with precious bacterial samples grown in growth-limiting conditions. Lastly, the findings of this study also suggest that good quality RNA is the major contributing factor to transcriptome data with excellent coverage and depth as the sequencing technology is known to be sensitive. A simple illustration in Fig. 6 outlines the workflow and the major conclusions drawn from this study.

## Additional file

10.1186/s13104-015-1726-3 **Table S1:** Representative transcript counts across replicates for each condition. Counts were consistent across the biological replicates that were sequenced, demonstrating the reproducibility and consistency of the RNA extraction technique.

## Abbreviations

RNA-Seq: RNA sequencing
RIN: RNA integrity number
sRNA: small RNA
ncRNA: non-coding RNA
