# Supplementary material for: Efficient extraction of small and large RNAs in bacteria for excellent total RNA sequencing and comprehensive transcriptome analysis
Source: BMC Res Notes. 2015 Dec 8;8:754. doi: 10.1186/s13104-015-1726-3 (PMC4673735; doi:10.1186/s13104-015-1726-3)
Supplement: Supplementary file 1 — 10.1186/s13104-015-1726-3 Table S1: Representative transcript counts across replicates for each condition. Counts were consistent across the biological replicates that were sequenced, demonstrating the reproducibility and consistency of the RNA extraction technique. [file 13104_2015_1726_MOESM1_ESM.docx]

**Additional File.**

**Table S1: Representative transcript counts across replicates for each condition.** Counts were consistent across the biological replicates that were sequenced, demonstrating the reproducibility and consistency of the RNA extraction technique.

| **Locus tag ID** | **Glucose-supplemented cells** | | **PE-supplemented cells** | |
| --- | --- | --- | --- | --- |
|  | **Replicate-1** | **Replicate-2** | **Replicate-1** | **Replicate-2** |
| PA4177 | 30 | 28 | 42 | 36 |
| PA2277 | 31 | 32 | 41 | 41 |
| PA3274 | 31 | 27 | 72 | 62 |
| PA0709 | 33 | 41 | 58 | 58 |
| PA0819 | 33 | 33 | 56 | 65 |
| PA1265 | 32 | 20 | 122 | 106 |
| PA0688 | 47 | 59 | 166 | 176 |
| PA3057 | 38 | 42 | 241 | 233 |
| PA2514 | 49 | 23 | 3485 | 3469 |
| PA1629 | 50 | 31 | 4506 | 4506 |
| PA3323 | 87 | 75 | 1100 | 1177 |
| PA1464 | 5199 | 4870 | 14251 | 13899 |
| PA0972 | 11237 | 11565 | 2450 | 2602 |
| PA0973 | 25235 | 26613 | 1819 | 1738 |
| PA1432 | 8412 | 7514 | 363 | 286 |
| PA1555 | 12201 | 11387 | 700 | 682 |
| PA1557 | 26086 | 27116 | 1721 | 1632 |
| PA3337 | 4437 | 4369 | 660 | 666 |
| PA3529 | 4794 | 4359 | 1224 | 1024 |
| PA4433 | 16199 | 16707 | 848 | 768 |
